# Seasonal influenza in children: Costs for the health system and society in Europe

**DOI:** 10.1111/irv.12991

**Published:** 2022-04-15

**Authors:** Leonardo Villani, Floriana D'Ambrosio, Roberto Ricciardi, Chiara de Waure, Giovanna Elisa Calabrò

**Affiliations:** ^1^ Section of Hygiene, University Department of Life Sciences and Public Health Università Cattolica del Sacro Cuore Rome Italy; ^2^ VIHTALI (Value in Health Technology and Academy for Leadership & Innovation) Spin‐Off of Università Cattolica del Sacro Cuore Rome Italy; ^3^ Department of Medicine and Surgery University of Perugia Perugia Italy

**Keywords:** children, costs, economic burden, Europe, pediatric influenza, vaccination

## Abstract

**Background:**

Pediatric influenza causes significant morbidity annually, resulting in an increased economic burden. Therefore, we aimed to summarize existing literature regarding the costs of pediatric influenza in Europe, paying particular attention to the direct and indirect costs considered in the economic evaluations. Knowing health and social costs of childhood influenza is essential to support value‐based health decisions to implement effective immunization strategies.

**Methods:**

We searched three databases for articles published to September 3, 2021. Eligible studies were those reporting the economic burden of influenza in the pediatric and youth population in European countries written in English language.

**Results:**

Overall, 2225 records were screened, and 9 articles were included. Costs estimates are different across countries and in the age groups considered. Direct costs per episode, whose major expense driver are hospitalizations and pediatric examinations, range from about €74 in Italy to €252 in Germany. Important variations are observed based on age, with the youngest group absorbing in some cases double the resources of the older ones such as (in Italy, in France and in Germany). Regarding indirect costs, workdays lost by parents resulted in higher costs for children <2 years and 2–5 years than those >5 years of age and their economic impact was variable reaching €251 per week in Germany.

**Conclusion:**

Evidence obtained in our review strengthened the awareness about the economic impact, in terms of direct and indirect costs, of pediatric influenza requiring, as a priority action in Europe, the implementation of influenza vaccination policies in this target population.

## 1 INTRODUCTION

Seasonal influenza causes significant morbidity and mortality annually, especially in vulnerable individuals such as children and the elderly, resulting in an increased economic burden on both health system and society.^1^ Indeed, every year, seasonal influenza epidemics result in about 1 billion cases, of which 3–5 million are severe cases, especially among the vulnerable groups, and about 250,000 to 500,000 deaths worldwide.^2^

In Europe, influenza causes 4–50 million symptomatic cases annually, about 15,000–70,000^3^ deaths and 150,000 influenza‐related^4^ hospital admissions. Specific vulnerable groups (the elderly, patients with chronic diseases and comorbidities, younger children [<5 years of age], and pregnant women) are at increased risk of developing severe illness, complications and dying from influenza.^5^ Anyone can get influenza, but infection rates are highest among the pediatric population (~20%–30% annually),^6^, ^7^ which presents a great risk of being infected due to limited pre‐existing immunity of children.^8^ Additionally, several studies have shown that young children play an important role in transmitting the influenza to their families and the wider community.^9^, ^10^, ^11^ The literature suggests that influenza vaccination of young children may reduce disease rates in non‐immunized individuals in the local community.^12^, ^13^, ^14^ Furthermore, although most influenza‐related complications and deaths occur among the elderly and people with underlying chronic conditions,^15^ children younger than 5 years of age are at high risk of developing serious influenza‐associated disease, with indirect impact on their siblings and parents.^8^ Actually, worldwide, each year approximately 870,000 children under 5 years of age have a hospitalization attributable to influenza, and it is estimated that between 28,000 and 111,500 deaths in this age group are attributable to influenza‐related causes, the vast majority of which occur in developing countries.^16^ Furthermore, it is estimated that the average annual rate of outpatient visits attributable to influenza are approximately 10, 100, and 250 times as high as hospitalization rates for children 0–5 months, 6–23 months, and 24–59 months of age, respectively.^17^

Among children, influenza has been considered serious only in those with chronic diseases and with medical conditions at higher risk of developing complications but in recent years the burden in healthy children has become increasingly explicit also thanks to the scientific evidence produced in the field of socio‐economic studies. Indeed, childhood seasonal influenza can impose substantial socio‐economic burden on healthcare system, families, and society in terms of hospitalizations, outpatient visits, medications, school absence, and missed workdays, either due to secondary illness in a caregiver or to the need to care for a sick child.^18^

An accurate assessment of the economic burden of influenza at the population level could guide policy decisions regarding influenza vaccination in children that is still widely debated in the European countries.^4^ As such, the aim of this systematic review is to summarize existing literature regarding the economic burden of childhood influenza per episode in Europe, paying particular attention to the type of costs (direct costs such as medical examinations, hospitalizations, drugs prescription, and indirect costs such as workdays lost by parents and children school leave) considered for the evaluation of the economic burden. In fact, investigating and knowing the healthcare and social costs of influenza in children is essential to support value‐based health decisions and to implement effective immunization strategies among children and youths in European Union (EU).

## 2 METHODS

### 2.1 Search strategy

A systematic review of the academic literature was conducted and reported according to the Preferred Reporting Items for Systematic Reviews (PRISMA).^19^ The literature search was performed by consulting three databases, namely, PubMed, Web of Science (WoS) and Scopus. The following search string was used on PubMed: “((“influenza, human”[MeSH Terms] OR “flu”[All Fields])) AND (“cost of illness”[MeSH Terms] OR “costs and cost analysis”[MeSH Terms] OR “economic burden”[All Fields]); this spelling was then adapted to WoS and Scopus. The following filters were applied: English language and target population (Child: birth‐18 years). The search was updated to September 3, 2021.

The articles records were entered in an Excel work sheet and screened according to the inclusion/exclusion criteria. A check for duplicates was performed; the selection was made firstly by reading titles and abstracts, and then the full texts.

### 2.2 Inclusion/exclusion criteria

All studies focused on the economic burden of influenza in the pediatric and youth population (age range 0–18 years) were considered potentially eligible. We included original articles and systematic reviews exclusively in English language and conducted in European Countries. No time limits were placed on the search, thus including all papers up to September 3, 2021. Studies describing the economic impact of influenza in populations aged >18 years and those concerning non‐European contexts were excluded. Narrative reviews, commentary, editorials, conference presentation, and citations not provided with full text were excluded as well as studies conducted in animals or in vitro.

### 2.3 Selection process and data extraction

Two researchers (L. V. and F. D'A.) independently screened titles and abstracts first and full texts afterwards. Any disagreement was resolved by discussion or by the involvement of a senior researcher (G. E. C.).

From the articles definitively included in the literature review, the following information were extracted: first author's name, publication year, country, study design, target population, setting, study duration and time, and main findings related to the economic burden of influenza, considering both direct and indirect costs.

When included, the systematic reviews were subjected to the snowballing process, using the bibliographic references and citations in the reviews in order to identify additional articles that met the inclusion criteria of this review.

## 3 RESULTS

**FIGURE 1:**
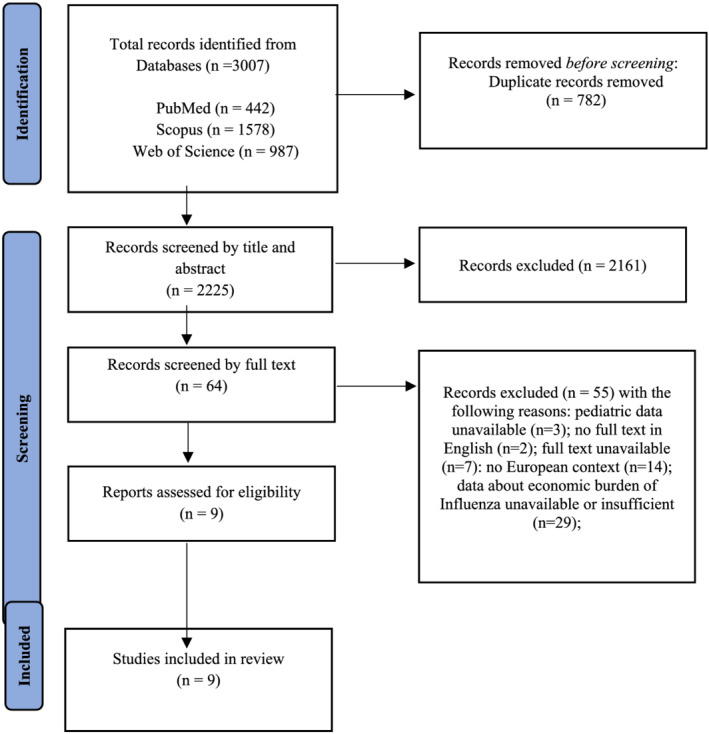
PRISMA statement flow diagram

The database search, after duplicates removal, brought a total of 2225 records. After an initial selection by titles and abstracts, 64 full‐text articles were selected. Following the inclusion and exclusion criteria, the screening resulted in the final inclusion of nine articles. Details about the study selection process are shown in the flowchart (Figure 1). Of the nine studies included in our systematic review, four (44.5%) were retrospective studies,^20^, ^21^, ^22^, ^23^ one was a case–control study (11.1%),^24^ two were prospective studies (22.2%),^25^, ^26^ and two (22.2%) systematic reviews.^8^, ^27^ No new studies were included after the snowballing process, because the two systematic review included studies that were already selected. The two systematic reviews reported international data and European information for the following countries: Italy, Germany, France, the Netherlands, Austria, Finland, Spain, and the United Kingdom.^8^, ^27^

The first systematic review published in 2012^8^ summarized influenza burden—especially in terms of health outcomes but also considering its economic impact—in children in Western Europe, emphasizing the significant direct impact of influenza on sick children and its indirect impact on their siblings and parents. Instead, the second revision of the 2018^27^ examined studies on the costs of Influenza‐Like Illness (ILI) in high‐income countries and considering all age groups of the population. Tables 1 and 2 report the details of each study, excluding the two systematic reviews. Among the seven primary studies included in our study, two (29%) were conducted in Germany,^22^, ^24^ two (29%) in Italy,^20^, ^25^ one (14%) in Sweden,^23^ one (14%) in France,^26^ and one (14%) in Belgium.^21^

**TABLE 1:** Main characteristics of the included studies about influenza among children and youths in the European context

First author, year and country	Target population (years old)	Study period	Study design and data sources	Methodology of costs evaluation
Lai 2011, Italy	0–14	1999–2008	Retrospective. Data regarding positive Influenza patients were collected from the Italian Influenza Surveillance System (CIRINET) in nine Italian regions.	In order to compare the economic burden of influenza in the different years, a conversion table from the Italian Statistics Institute was used to equate the purchasing power of the Euro in the different years considered.
Esposito 2011, Italy	0–13, with the following age groups: <2 years 2–5 years 6–13 years	2008–2009	Prospective. Study on children with ILI, trough the Pedianet network. Pedianet is an Italian independent network of more than 400 family pediatricians (FP) established in 1998 to collect relevant data for clinical and epidemiological research.	Direct and indirect costs were analyzed from the individual and social perspective. Costs are calculated per patient. Costs related to drug prescriptions are obtained from the Italian Directory of Medicines and manufactures, 2009. The costs of medical examinations are obtained from the 1997 National Tariff Nomenclator, adapted to 2008. The costs of hospitalizations are based on the DRGs of the Veneto Region. The indirect costs are calculated based on the productivity rate provided by the Bank of Italy 2002 divided by 220 working days.
Crott 2014, Belgium	0–17, with the following age groups: 0–4 years 5–17 years	2002–2007	Retrospective. Data were obtained from a database of all acute inpatient stays of 11 hospitals using the Belgian Minimal Hospital Summary Data.	The costs considered are reimbursement costs including records for only those patients who are covered by the Belgian National Health insurance.
Silva 2014, France	0–14 with the following age groups: 0–4 years 5–14 years	2010–2011	Prospective. Data were obtained from the Influenza B in General Practice (IBGP) study.	Costs were assessed and calculated from the French Health Insurance perspective, based on a micro‐costing approach that analyzed the reimbursed fees and the GROG network (Groupes Régionaux dObservation de la Grippe). Costs were calculated per patient and an estimation of the costs in whole pediatric French population was obtained by applying the cost‐related information to the national incidence estimates for influenza B.
Ehlken 2015, Germany	0–16	2010–2012	Retrospective. Data were obtained from a longitudinal electronic medical records (EMR) database including patient‐level data from the physician‐practice data systems of office‐based physicians and pediatricians.	Direct costs were considered from payer and patients perspective. The analysis was based on resource utilization data. Indirect costs were considered from societal perspective and based on gross wage data and the number of persons in dependent employment (source: German Federal Statistical Office 2012).
Rahmqvist 2016, Sweden	2–17	2005–2012	Retrospective. Data about ILI in pediatric population were obtained from the regional healthcare register of a Swedish region.	Medical costs for inpatient care were priced using the Cost Per Patient module. Drug prescriptions data and costs were retrieved from the National Bord of Helth and Welfare. Indirect costs of ILI were based on the number of reimbursed VAB days (temporary prenatal benefits for staying home to take care of child).
Scholz 2019, Germany	0–17, with the following age groups: 0–1 years 2–5 years 6–9 years 10–17 years	2012–2014	Case–control. Claims data were obtained from >8 million insured of a large German sick‐ness fund were analyzed, covering a 3‐year period (2012–2014).	Inpatient costs were determined by the payments of the sickness fund to the hospitals. Indirect costs were retrieved from the claims data considering the number of days absent from work and calculated only for employed persons.

**TABLE 2:** Main findings, direct and indirect costs of the included studies

First author, year	Cost drivers	Main findings	Direct costs (mean ± standard deviation)		Indirect costs (mean ± standard deviation)	
			Children ≤5 years	Children ≥5 years	Children ≤5 years	Children ≥5 years
Lai 2011, Italy	Total costs of influenza	Costs are calculated per episode in each season.	**‐**	**‐**	**‐**	**‐**
		**Children 0–14 years:**				
		1999/00: €207.73				
		2000/01: €210.54				
		2001/02: €213.00				
		2002/03: €213.00				
		2003/04: €214.64				
		2004/05: €216.68				
		2005/06: €218.73				
		2006/07: €219.14				
		2007/08: €217.30				
		2008/09: €211.57				
		2009: €204.61				
		2009/10: €204.61				
		Average: €205.09				
Esposito 2011, Italy	**Direct costs** ‐ Medical examinations ‐ Drug prescriptions ‐ Hospitalizations **Indirect costs** ‐ Work days lost by parents	Costs are calculated per episode. **Children <2 years**: €153.2 ± 72.8 **Children 2–5 years**: €148.1 ± 83.1 **Children 6–13 years**: €73.9 ± 41.9 Influenza‐ positive ILI was 32% more expensive than influenza‐negative ILI. Moreover, Influenza A cases were significantly more expensive than influenza B cases (€142.60 ± 74.3 vs. €72.80 ± 53.3).	**Children <2 years** Pediatric examinations: €33.5 ± 5.6 Antibiotic use: €3.2 ± 3.9 Antipyretic use: €2.4 ± 1.9 Hospitalization: €40.8 ± 238.8 **Children 2–5 years** Pediatric examinations: €32.9 ± 4.2 Antibiotic use: €4.0 ± 4.6 Antipyretic use: €2.3 ± 2.2 Hospitalization: €23.9 ± 268.9	**Children 6–13 years** Pediatric examinations: € 33.0 ± 2.5 Antibiotic use: €3.3 ± 3.9 Antipyretic use: €2.1 ± 2.5 Hospitalization: €11.5 ± 153.4	**Children <2 years** Working days lost by mothers: €46.7 ± 96.4 Working days lost by fathers: €26.6 ± 90.4 **Children 2–5 years** Working days lost by mothers: €55.6 ± 106.7 Working days lost by fathers: €29.4 ± 111.4	**Children 6–13 years** Working days lost by mothers: €19.8 ± 49.6 Working days lost by fathers: €4.2 ± 39.1
Crott 2014, Belgium	**Direct costs** Hospitalizations	Costs are calculated per episode. Children represent the second largest group after the elderly with the highest number of hospitalizations and they are responsible for about 20% of the total costs.	**Children <5 years** Hospitalization (pneumonia and influenza): €2207 ± 1658 Hospitalization with respiratory and circulatory complications: € 2617 ± 2314	**Children 5–17 years** Hospitalization (pneumonia and influenza): €2390 ± 1845. Hospitalization with respiratory and circulatory complications: € 2519 ± 2083	‐	‐
Silva 2014, France	**Direct costs** ‐ Medical examinations ‐ Hospitalizations ‐ Influenza Vaccine cost (excluding administration) ‐ Drug prescriptions ‐ Additional tests costs (out of hospital) **Indirect costs** ‐ Work days lost by parents ‐ Children school leave	Costs are calculated per episode. **Children 0–4 years** Total costs: €70 ± 261.60 **Children 5–14 years** Total costs: €50 ± 194.80	**Children 0–4 years** Initial consultation at medical office (GP or pediatrician): €20.70 ± 1.70 Vaccine: €0.30 ± 1.30 Follow‐up consultations at medical office (GP): €3.30 ± 9.90 Follow‐up at home (GP): €0.50 ± 3.60 Emergency services: €0.00 ± 0.00 Hospitalization: €36.0 ± 254.30 Pharmaceutical spending: €6.50 ± 10.30 (of which antibiotics: €0.80 ± 2.20, antivirals: €0.70 ± 1.50, other drugs: €5.00 ± 9.20, additional tests: €2.80 ± 11.40)	**Children 5–14 years** Initial consultation at medical office (GP or pediatrician): €17.10 ± 1.60 Vaccine: €0.20 ± 1.10 Follow‐up consultations at medical office (GP): €3.50 ± 9.80 Follow‐up at home (GP): €0.50 ± 4.80 Emergency services: €1.20 ± 6.50 Hospitalization: €19.80 ± 188.50 Pharmaceutical spending: €5.50 ± 8.80 (of which antibiotics: €0.60 ± 1.80; antivirals: €0.80 ± 1.80; other drugs: €4.10 ± 8.50; additional tests: €2.20 ± 2.40)	**Children 0–14 years** Working days lost by parents: 2.8 days School days lost by children: 5.6 days	
Ehlken 2015, Germany	**Direct Costs** ‐ Medical examinations ‐ Hospitalizations ‐ Drug prescriptions	Costs are calculated per episode. **Children 0–16 years** Total costs: €102 ± 224 Total cost per influenza/ILI episode without complications: €55.00 Total cost per influenza/ILI episode with at least one complication: €149.00 Physician visits: €35.00Referrals to other physicians: €1.00Hospitalization with relevant diagnoses: €17.00 Pharmaceutical spending: €13.00	**Children <5 years** Total cost per influenza/ILI episode: €124.00	**Children 5–16 years** Total cost per influenza/ILI episode: €96.00	‐	‐
Rahmqvist 2016, Sweden^a^	**Direct costs** ‐ Medical examinations ‐ Hospitalizations ‐ Drug prescriptions **Indirect costs** ‐ Work days lost by parents	Costs are calculated per season. The total annual costs of ILI amount to €400 400 per 10 000 children.	Of the total costs, primary care generates 63.9%, outpatient activities 25.5%, and hospitalizations 10.6%		Indirect costs associated with the loss of parental productivity correspond to 2.2 to 2.6 million Euros per year for 10,000 children. Thus, annual indirect costs were 5.2 to 6.2 times greater than direct costs (2.2–2.6 million vs. 0.42 million per 10,000 children per year).	
Scholz 2019, Germany	**Direct costs** ‐ Hospitalizations ‐ Drug prescriptions **Indirect costs** ‐ Work days lost by parents	Costs are calculated per episode. Total costs: **Children 0–1 years**: €251.91 **Children 2–5 years**: €103.21 **Children 6–9 years**: €64.88 **Children 10–17 years**: €76.18	**Children 0–1 years** Hospitalization^b^: €68.76 Complications of otitis media and pneumonia^b^: €3.68 and €123.87 Outpatient treatments: €47.99 ± 298.04 Pharmaceutical spending: €7.60 ± 21.33 **Children 2–5 years** Hospitalization^b^: €21.29 Complications of otitis media and pneumonia^b^: €0.47 and €27.52 Outpatient treatments: €46.24 ± 391.38 Pharmaceutical spending: €7.75 ± 28.37	**Children 6–9 years** Hospitalization^b^: €13.59 Complications of otitis media and pneumonia^b^: €1.30 and €8.49 Outpatient treatments: €36.95 ± 400.85 Pharmaceutical spending: €7.16 ± 58.29 **Children 10–17 years** Hospitalization^b^: €7.79 Complications of otitis media and pneumonia^b^: €0.15 and €4.10 Outpatient treatments: €55.62 ± 447.34 Pharmaceutical spending: €8.52 ± 53.60	‐	**Children 10–17 years** Costs related to the loss of parental productivity amounted to €251.36 per week

^a^ Direct and indirect costs are expressed per 10,000 inhabitants.

^b^ Standard deviation not available.

Ehlken et al. in 2015 and Scholz et al. in 2019 analyzed the costs of pediatric influenza in Germany. The first study^22^ reported an average total cost of ILI per capita in the years 2010–2012 of €106.00 for children aged 0–16 years (€36.00 for medical visits, €17.00 for hospitalizations, €13.00 for drug treatment, €30.00 for outpatient medical visits, and €10.00 for indirect costs attributable to parental loss of productivity). The costs associated to children <5 years (€124.00) were higher than those in children aged 5–16 years (€96.00).

The second study^24^ analyzed the costs associated with influenza during the period 2012–2014 in the pediatric population, stratified into four age groups: 0–1, 2–5, 6–9, and 10–17 years. On average, the total direct costs per capita amounted to €251.91, €103.21, €64.88, and €76.18 for 0–1, 2–5, 6–9, and 10–17 years group, respectively. The 0–1 years group absorbed the largest amount of resources, especially for costs related to hospitalizations and treatment of complications. Drugs spending was higher in the 10–17 years group (€8.52) followed by the 2–5 years group (€7.75), the 0–1 years group (€7.60) and the 6–9 years group (€7.16). Considering the indirect costs, the study focused only on the 10–17 years group, with costs related to loss of productivity of € 251.36 per week.

Lai et al.^20^ assessed the economic burden of ILI in children aged 0–14 years in nine Italian regions during 11 influenza seasons (months October–April) from 1999/00 to 2009/10, analyzing data from the National Italian Influenza Surveillance System (INFLUNET). The average estimated cost of ILI seasonal outbreaks was €156,555,103, with a maximum value reported in the 2004/05 season (€270,897,733) and a minimum in the 2005/06 season (€40,997,490). As the study also reported the number of influenza cases for each season, we were able to calculate the total cost per episode, with values ranging from a minimum of €204.61 (season 2009/10) to a maximum of €219.14 (season 2006/07). On average, the per‐episode cost was equal to €205.09 during the entire period considered. The study did not assess the direct and indirect costs of influenza, but only the global economic burden of influenza in Italy.

Esposito et al.^25^ evaluated the clinical and economic burden of influenza in healthy children aged <14 years in the 2008/09 season. A total of 21 986 children, 6988 of whom had ILI and underwent nasopharyngeal swabbing, were enrolled in this prospective study. Laboratory‐confirmed influenza cases were associated with higher costs than influenza virus‐negative ILI (+32%), with an average total cost per case of €131.70 and €89.40, respectively.

Influenza A cases were significantly more expensive than influenza B cases (€142.60 vs. €72.80). Moreover, pediatric visits (on average €33.00) and hospitalizations (on average €22.40) contributed most to the average total cost of influenza. Analyzing the different age groups, children <2 years and between 2 and 5 years absorbed almost twofold resources than those >5 years (€153.20, €148.10, and €73.90, respectively). In particular, workdays lost by mothers resulted in higher indirect costs for children <2 years and 2–5 years than those >5 years of age (€46.70, €55.60, and €19.80, respectively).

The study of Rahmqvist^23^ analyzed both direct and indirect healthcare costs associated with seasonal influenza/ILI in children 2–17 years of age, from 2005 to 2012. The total annual costs for ILI amounted to €400,400 per 10,000 children aged 2 to 17 years. Primary care, outpatient activities and hospitalizations accounted for 63.9%, 25.5%, and 10.6% of the costs, respectively. Considering the costs related to drug consumption, antibiotics, nasal solutions, cough medicines, and respiratory drugs were considered, with a total cost of €23,200 per 10,000 inhabitants each year. In addition, the costs of these drugs increased by an average of 30.3% (€850.00 per week per 10,000 inhabitants) per week from the pre‐flu season to the peak of the flu season. Costs related to the consumption of these four classes of drugs corresponded to 5.8% of total direct healthcare costs. Moreover, it was estimated a loss of productivity of €2.2–2.6 million per year for 10,000 children aged 2 to 17 years.

In France, Silva et al.^26^ assessed the economic impact in terms of direct healthcare costs of type B influenza in children <14 years of age, during the season 2010–2011. The average cost was higher in the group 0–4 years, with a value of €70.00 per capita compared with €50.00 for the 5–14 years age group. Moreover, it was reported a cost for hospitalizations and for the first visits to the pediatrician of €36.00 and €19.80 and €20.70 and €17.10 (0–4 years and 5–14 years, respectively).

The study conducted by Crott et al.^21^ considered the costs associated with hospitalizations for influenza in Belgium in children <7 years old in the period 2002–2007. The study analyzed the possible complications and estimated an average cost per hospitalization due to pneumonia and influenza of €2207.00 (±€1658.00) for the 0–4 years age group and €2390.00 (±€1845.00) for children aged 5–17 years. Considering the complications, the average cost per hospitalization amounted to €2617.00 (±€2314.00) for the 0–4 years age group and €2519.00 (±€2083.00) for children aged 5–17 years.

## 4 DISCUSSION

Our review summarizes the currently available evidence on the economic burden of influenza among European children and youths. In particular, it is evident the impact (both in terms of direct and indirect costs) of influenza borne by families and therefore by society, especially in children <5 years old. Costs estimates are different across countries and in the age groups considered, making comparison difficult. These differences may reflect study‐specific characteristics, including study design, different study populations, and types of costs included in the analysis.

It is well known that the clinical burden of influenza is associated with a significant economic impact linked both to medical visits, hospitalizations and drug use, as well as to the indirect consequences, namely the loss of productivity.^27^ In fact, the studies included in this systematic review assessed the economic burden of influenza considering, in most cases, both the perspective of the health system and of society. They showed that influenza is generally associated with substantial socio‐economic consequences on families, healthcare services and society although the clinical and economic burden of influenza vary with the severity of the influenza season.^20^

The costs associated with influenza also vary in relation to the influenza virus type. In fact, according to Esposito et al.,^25^ influenza A cases were significantly more expensive than influenza B cases and this could be due to the fact that influenza A virus appears to cause more severe diseases than influenza B virus.^25^, ^28^ Eventually, influenza in children aged <2 and 2–5 years was significantly more expensive than in children aged >5 years. The difference was mainly associated with the indirect costs due to parents' lost working days and could be justified by the evidence suggesting that influenza A occurs more frequently among children <4 years of age than in older children.^28^, ^29^

Therefore, it is evident that influenza in children aged <5 years is more expensive than in older children.^25^ In this age group, the higher costs are linked both to an increase in direct costs for hospitalizations, medical visits and medications, and, above all, to indirect costs linked to the missed workdays, either due to secondary illness in caregivers, namely, parents, or their need to care for a sick children.^21^, ^22^, ^24^, ^25^, ^26^ As regards direct costs, those related to hospitalizations have an important impact and are higher for children aged <1 years than in older.^24^ Hospitalization costs increase in the case of flu complications especially in younger children. Scholz et al.^24^ showed higher costs in children with pneumonia and aged <1 years. Also Ehlken et al.,^22^ showed that total direct costs per influenza/ILI episode with at least one complication was approximately 2.7 higher than those per episode without complications.

Influenza directly causes viral and secondary bacterial pneumonia, upper respiratory tract infections such as sinusitis and otitis media and can facilitate coinfection with other respiratory pathogens. Furthermore, influenza can exacerbate underlying chronic conditions like diabetes, pulmonary and cardiovascular diseases.^21^ Evidence suggests that influenza complications occur more frequently and are more severe in vulnerable groups such as the elderly and younger children (<5 years of age).^15^ In addition to the common flu complications, young children are also susceptible to further complications of the non‐respiratory tract, notably cardiovascular ones.^30^ Although less common, hospitalization for both respiratory and non‐respiratory influenza related complications could be expensive.^21^ However, the costs of hospitalizations are not the only ones that affect the economic burden of influenza. In the absence of complications, primary care manages most visits for influenza and ILI. Actually, primary care generates 63.9%, of total annual costs of ILI and the costs for pediatric visits are higher in children aged <5 years vary from €21.00^26^ to €33.00^25^ approximately. Another important information is related to the use of antibiotics. It is estimated^25^ that more than 40% of children with influenza receives antibiotics and they are less frequently prescribed to children with laboratory‐confirmed influenza compared with flu‐negative patients. However, inappropriate prescriptions of antibiotics to laboratory‐confirmed influenza patients were reported to be as high as 30% in the United States.^31^ Not only these practices prompt unnecessary costs to the healthcare system but they may also favor the emergency of drug‐resistant infections in the long term.^27^ Considering this type of information in pharmacoeconomic studies could provide useful evidence for decision and policymakers.

In different studies included in our systematic review, indirect costs were shown to contribute most to the average total cost of influenza.^23^, ^24^, ^25^ In particular, workdays lost by parents resulted in higher indirect costs for children <2 years and 2–5 years than those >5 years of age and are higher for workdays lost by mothers.^25^

Furthermore, the role of children in influenza transmission to their elderly contacts was demonstrated by Ghendon et al.^32^ when the rate of ILI was 3.4 times lower in the elderly contacts of immunized children than in contacts of the control group.

The indirect economic burden associated with secondary infection of family contacts of sick children, parental illness, lost working days and flu transmission in the workplace should be further and properly evaluated.

That said, considering that, for example, 0.3%–9.8% of children aged 0–14 years present to a physician with ILI during 2002/2003–2008 and 47%–83% are caused by influenza virus in the average season,^33^ it is evident that influenza is a public health major issue and vaccination remains the cornerstone for preventing influenza and its consequences in all age groups, and for reducing its clinical and economic burden on healthcare systems, and communities.^4^, ^34^ For this reason, different practical indications for children immunization were released and applied worldwide. In this context, from 2012 the WHO Strategic Advisory Group of Experts (SAGE) recommended immunization of children younger than 5 years old, particularly those aged <2 years, in order to protect children and, indirectly, unvaccinated household members (herd immunity) and community contacts.^35^ This recommendation, starting in the season flu 2017–2018, was also applied in some member States of the EU such as Finland, Latvia, Malta, Slovakia, Slovenia, and the UK. However, in most European countries influenza vaccination in healthy children is still widely debated and only a minority of industrialized countries include universal influenza vaccination in their pediatric immunization programs.^4^ Indeed, allocating resources to influenza vaccination campaign is not always considered a priority for the National Health Authorities, albeit its recommendation free of charge for the whole pediatric population should be encouraged: improving children's health, vaccination also contributes to enhanced societal economic well‐being with significant cost savings for all the levels of government.^36^, ^37^, ^38^, ^39^, ^40^ Childhood vaccination may also result in societal benefits, reducing onward transmission in households, preventing premature disability or death among parents and grandparents and gaining number of quality‐adjusted life‐years.^41^, ^42^

To achieve this goal, there is the need for aligning and sharing of international guidelines, taking into account the broad value of vaccines^43^, ^44^ (personal, technical, allocative, and social value) and their short‐ and long‐term benefits, and encouraging greater involvement of public health and vaccination experts, policy makers and citizens.

Our review provides evidence about the economic burden of influenza in children and youths in the European context, underlining the direct and indirect costs associated with infection in this target population and, consequently, how flu vaccination may protect not only vaccinated children but also indirectly their social contacts and the population as a whole. Furthermore, our review highlights the need for new evidence on the overall economic burden of influenza in children that takes into account both the healthcare and social perspectives in the construction of economic models. Comprehensive information on disease costs can support value‐based decision making as recently proposed by the Expert Panel on Effective Ways of Investing in Health (EXPH) of the European Commission.^45^

Nevertheless, several limitations should be considered when interpreting our results. Only articles published in English were included, which might have led to fail identifying all the available evidence on the topic. Furthermore, a quality assessment of the included articles was not performed, and we could not assess the methodological correctness of the included studies. However, in our opinion, this does not impair our work as we wanted to provide an overview of the economic burden of childhood influenza in Europe without addressing the robustness of methods used to do it. Eventually, the heterogeneity of studies in terms of study‐specific characteristics, including study design, study populations, and types of costs considered and their limited transferability from one country to another prevent to issue definite estimates of the economic burden of childhood influenza. Furthermore, in the included studies, no detailed information is available on the number of consultations, days of admission, number of ICU admission, and so on, and this aspect also limits the possibility of comparisons on the consumption of flu‐related resources.

However, the overview that we have provided pinpointed several meaningful aspects, namely age‐dependent differences in costs, contribution of indirect costs to the total cost, which can help the appraisal of the value of influenza vaccination in decision‐making.

## 5 CONCLUSION

This review summarized the evidence about the economic burden of influenza in children and provided some interesting information on costs occurred by influenza. However, a relevant finding that emerged from our review is the scarcity of evidence on the costs associated with influenza among children in Europe as well as the lack of standardized methods for their evaluation and, therefore, the difficulty of analyzing and comparing them comprehensively. Complete information on pediatric influenza costs might be of interest for decision makers to ensure better resources allocation in prevention of vaccine preventable diseases and to implement value‐based immunization strategies.

## AUTHOR CONTRIBUTIONS

**Leonardo Villani:** Data curation; formal analysis; methodology. **Floriana D'Ambrosio:** Data curation; formal analysis; methodology. **Roberto Ricciardi:** Conceptualization; project administration; supervision; validation. **Chiara de Waure:** Validation. **Giovanna Elisa Calabrò:** Conceptualization; data curation; formal analysis; methodology; writing; validation; supervision.

### PEER REVIEW

The peer review history for this article is available at https://publons.com/publon/10.1111/irv.12991.

## Data Availability

The data that support the findings of this study are available from the corresponding author upon reasonable request.
